# Indispensable Adaptability: Adjusting to the Rapid Changes of the Scientific World

**DOI:** 10.1590/0100-6991e-20233693EDIT01-en

**Published:** 2023-11-23

**Authors:** Andreia Cristina Feitosa do Carmo, Julia Castro Neves, Gerson Alves Pereira, Ramiro Colleoni

**Affiliations:** 1 - Universidade Federal de São Paulo, Biblioteca do Campus São Paulo - São Paulo - SP - Brasil; 2 - Colégio Brasileiro de Cirurgiões, Departamento de Publicações - Rio de Janeiro - RJ - Brasil; 3 - FOB/USP, Curso de Medicina de Bauru - Bauru - SP - Brasil; 4 - Universidade Federal de Sao Paulo, Escola Paulista de Medicina - São Paulo - SP - Brasil

## EDITORIAL

We are immersed in an era of accelerated changes in the process of preparing and evaluating scientific publications. The propagation of scientific knowledge has undergone significant transformations, largely driven by the increasing integration of information technology. A notable change in this context is the transition towards open science, a movement that began in the 1990s and emphasizes the sharing of knowledge in a transparent and ethical manner, making it accessible to all through communication networks. With innovation as its pillar, this movement is driving a profound overhaul of conventional publishing standards.

An essential component of open science is open access (OA), which has been adopted over the last few decades by leading scientific publishers. OA allows journal content to be freely accessible to anyone with Internet access.

Adapting to these changes is essential if the Journal of the Brazilian College of Surgeons (JBCS) is to maintain its relevance and continue to contribute to national and international research. In this context, we would like to highlight five areas of strategic focus for the JBCS:

- Inclusion and Diversity: we are committed to cultivating an inclusive environment that values diversity of perspectives, voices and areas of research, contributing to the richness of scientific discourse.
- Quality and Rigor: we seek to raise the standards of quality and rigor in the selection and review of manuscripts, ensuring that each publication meets the highest standards of scientific excellence.
- Transparency and Reproducibility: we are committed to promoting transparency in publication practices and encouraging the reproducibility of studies, so that each article makes a solid and reliable contribution to the construction of scientific knowledge.
- Engagement and Communication: we are looking to emerging technologies and social media platforms to increase engagement with the scientific community and the general public, encouraging dissemination and dialog about published research.
- Sustainability and Innovation: We will continue to explore sustainable publishing models and embrace continuous innovation to ensure RCBC’s longevity and relevance in the ever-evolving landscape of scientific publishing.

By prioritizing these areas, JBCS will remain well positioned to navigate current and future changes in the field of scientific publishing, continuing to provide quality articles for the dissemination of high-quality research.

Furthermore, it is imperative that the scientific community remains up-to-date and proactive in the face of new developments in the publishing sphere. The advancement of science is a collaborative enterprise, and as such requires the involvement and contribution of all stakeholders - including researchers, professionals, students, editors and reviewers.

Ethics and integrity must also be paramount in our efforts. As we embrace the new possibilities offered by open science and open access, we must also ensure that rigor, honesty and accountability are at the forefront of our publishing practices.

JBCS is committed to serving not only as a repository of knowledge, but also as a facilitator of academic dialog and a promoter of scientific advancement. Through a combination of continuous innovation, commitment to excellence and adherence to the highest ethical standards, we seek to make a significant contribution to the development of the scientific community.

In conclusion, we are grateful for the continued support and contributions of the scientific community and our reviewers, who are vital to our mission. Their dedication to advancing knowledge is what enables our success, which is collective. We invite everyone to join us on this journey of adaptation and growth, facing the challenges and seizing the opportunities presented by an increasingly interconnected and dynamic world.

Through collaboration, integrity and innovation, let’s together strengthen the foundation on which science, especially Brazilian science, can thrive and serve as a catalyst for the progress and well-being of society.
